# The genome sequence of *E. coli *W (ATCC 9637): comparative genome analysis and an improved genome-scale reconstruction of *E. coli*

**DOI:** 10.1186/1471-2164-12-9

**Published:** 2011-01-06

**Authors:** Colin T Archer, Jihyun F Kim, Haeyoung Jeong, Jin Hwan Park, Claudia E Vickers, Sang Yup Lee, Lars K Nielsen

**Affiliations:** 1 Australian Institute for Bioengineering and Nanotechnology, Cnr Cooper and College Rds, The University of Queensland, St Lucia, Queensland 4072 Australia; 2 Industrial Biotechnology and Bioenergy Research Center, Korea Research Institute of Bioscience and Biotechnology, 111 Gwahangno, Yuseong-gu, Daejeon, Korea; 3 Department of Chemical and Biomolecular Engineering (BK21 program) and Center for Systems and Synthetic Biotechnology, Institute for the BioCentury, KAIST, 335 Gwahangno, Yuseong-gu, Daejeon 305-701, Republic of Korea

## Abstract

**Background:**

*Escherichia coli *is a model prokaryote, an important pathogen, and a key organism for industrial biotechnology. *E. coli *W (ATCC 9637), one of four strains designated as safe for laboratory purposes, has not been sequenced. *E. coli *W is a fast-growing strain and is the only safe strain that can utilize sucrose as a carbon source. Lifecycle analysis has demonstrated that sucrose from sugarcane is a preferred carbon source for industrial bioprocesses.

**Results:**

We have sequenced and annotated the genome of *E. coli *W. The chromosome is 4,900,968 bp and encodes 4,764 ORFs. Two plasmids, pRK1 (102,536 bp) and pRK2 (5,360 bp), are also present. W has unique features relative to other sequenced laboratory strains (K-12, B and Crooks): it has a larger genome and belongs to phylogroup B1 rather than A. W also grows on a much broader range of carbon sources than does K-12. A genome-scale reconstruction was developed and validated in order to interrogate metabolic properties.

**Conclusions:**

The genome of W is more similar to commensal and pathogenic B1 strains than phylogroup A strains, and therefore has greater utility for comparative analyses with these strains. W should therefore be the strain of choice, or 'type strain' for group B1 comparative analyses. The genome annotation and tools created here are expected to allow further utilization and development of *E. coli *W as an industrial organism for sucrose-based bioprocesses. Refinements in our *E. coli *metabolic reconstruction allow it to more accurately define *E. coli *metabolism relative to previous models.

## Background

*Escherichia coli *is a model prokaryotic organism, an important pathogen and commensal, and a popular host for biotechnological applications. Among thousands of isolates, only four strains (the common laboratory strains K-12, B, C, and W) and their derivatives are designated as Risk Group 1 organisms in biological safety guidelines [1,2]. A fifth strain, *E. coli *Crooks (ATCC 8739), has also been used extensively in laboratories for over 70 years [3-5]; more recently, it has been used as a host for industrial biochemical production [6-8]. There have been no reported cases of the strain being pathogenic, suggesting that it is generally safe. When it was sequenced in 2007, ATCC 8739 was designated as a C strain [6], however, it is in fact a Crooks strain [4] and recent publications have reflected this correction [9,10]. Of these five safe strains, K-12 [11], B [12] and Crooks [GenBank:CP000946] have been sequenced, but C and W have not.

*E. coli *W (ATCC 9637) was originally isolated from the soil of a cemetery near Rutgers University around 1943 by Selman A. Waksman, around the same time he and Alan Schatz discovered streptomycin (Eliora Ron, personal communication). Waksman coined the term 'antibiotic', and his discovery of streptomycin (and many other antibiotics) led to him being awarded the Nobel Prize in Physiology or Medicine in 1952. The strain was termed "Waksman's strain" or "W strain" because it showed the highest sensitivity to streptomycin compared to other isolated *E. coli *strains in Waksman's collection (Eliora Ron, personal communication).

The first reported use of W was as the standard *E. coli *strain in the assay for sensitivity to streptomycin and other antibiotics [13]. Bernard Davis, a prominent microbiologist from Harvard Medical School, developed a large auxotrophic mutant library from the strain [14] using his penicillin-based selection technique [15]. One of these mutants, vitamin B-12 auxotroph 113-3 (ATCC 11105), is well known as a production strain for penicillin *G acyclase* (PGA) [16] and for studies of aromatic compound degradation in bacteria [17]. It has also recently been discovered that the popular ethanol-producing strain KO11 [18] is a W strain rather than a B strain as previously thought [19]. Both W and KO11 have been engineered for the production of several chemicals, including ethanol [18,20,21], poly-3-hydroxybutyrate[22], lactic acid [23] and alanine [19]. The W strain has several properties that make it a preferred strain for industrial applications. It produces low amounts of acetate even without tight sugar control, and can be grown to high cell density during fed-batch culture with relative ease [22]. It also has good tolerance for environmental stresses such as high ethanol concentrations, acidic conditions, high temperatures and osmotic stress [24,25]. It is a very fast growing strain; its superior growth rate on LB medium compared to classical K-12-derived strains has led to it being developed as a lab cloning strain [27]. These combined characteristics make W extremely attractive as a production strain. Significantly, W is the only safe *E. coli *strain which can utilize sucrose as a carbon source, and it grows as fast on sucrose as it does on glucose [22,27,28]. Sucrose is emerging as a preferred carbon source for industrial fermentation: life cycle analysis demonstrates that sucrose from sugarcane has a superior performance when compared to glucose from starch [29].

Modern development of good production strains entails application of metabolic engineering principles. Increasingly, metabolic engineering relies on a systems biology approach [30]; a key aspect of this approach is the integration of a metabolic model (genome-scale model, GEM). The first step in developing a GEM is to build an *in silico *genome-scale reconstruction (GSR) derived from the organism's genome sequence. In this paper, we present the complete genome sequence, detailed annotation of *E. coli *W. Comparative genome analyses were performed among safe *E. coli *strains and group B1 commensal/pathogenic *E. coli *strains. In addition, a comprehensive, W-specific GSR was developed to underpin construction of a GEM for engineering industrial production strains.

## Results and Discussion

### Annotation and comparative analysis with other safe laboratory strains

**Figure 1:**
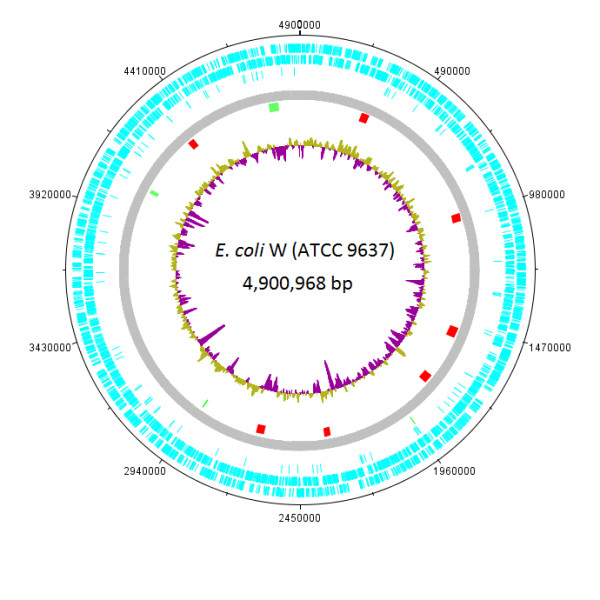
**Circular map of the E. coli W chromosome**. The outer circle shows position in bp. The second, third and fourth circles (blue) show forward ORFs, reverse ORFs, and pseudogenes, respectively. The fifth circle (green) shows pseudoknots. The seventh circle shows large mobile elements (see Table 2 for details); pLEs are in green and prophages are in red. The inner circle shows a plot of G+C content, with purple being G+C and tan being A+T.

A combination of Roche/454 pyrosequencing, fosmid end sequencing and Sanger sequencing was used to obtain the complete genome sequence of *E. coli *W (ATCC 9637). The W genome consists of a circular chromosome [Genbank: CP002185] (Figure 1) and two plasmids, pRK1 [Genbank: CP002186] and pRK2 [Genbank: CP002187]. Detailed results of genome analysis can be found in Table 1. At 4,901 Kbp, the chromosome of *E. coli *W is the largest of all the sequenced safe laboratory strains. Comparison with available *E. coli *genome sequences in GenBank demonstrated that it is similar in size to the commensal *E. coli *strain SE11 (4,888 Kbp) [31], but smaller than most sequenced pathogenic strains. A total of 4,764 chromosomal genes (including 82 non-coding RNA genes) were predicted using Prodigal [32] and Glimmer[33]; these genes cover 89% of the chromosome.

**Table 1:** Summary of genome features in safe strains.

	pRK1	pRK2	W	K-12	B	Crooks
Accession & Version	CP002186	CP0021857	CP002185	U00096.2	CP000819.1	CP000946.1
Chromosome size (Kbp)	102.5	5.36	4901	4640	4630	4746
G+C content	49.95	46.03	50.84	50.78	50.77	50.87
genes (pseudogenes)	117 (1)	16 (0)	4764 (91)	4493 (177)	4383 (67)	4409 (82)
CDSs	114	15	4482	4149	4209	4200
structural RNAs	3	1	191	172	107	128
rRNAs	0	0	22	22	22	22^a^
tRNAs (pseudo)	0 (0)	0 (0)	87	89 (3)	85 (0)	87 (1)
other ncRNAs (pseudo)	2 (0)	1 (0)	82	61 (2)	ND	19
Large Mobile Elements	0	0	10	10	11	9
Prophage regions	0	0	7	8	10	8
Integrative Elements	0	0	3	2	1	1
IS elements (pseudo)	2 (0)	0 (0)	18 (6)	41 (13)	50 (12)	39 (15)
LPS core type	-	-	R1	K-12	R1 (IS1::*waaT*)	R1
O antigen	-	-	O6	O16 (IS5::*wbbL*)	O7 (IS1::*wbbD*)	O146 (IS1::*wbwW*)
H antigen			H49	H48	-	ND
K antigen	-	-	-	-	K5 (IS1::*kfiB*)	-
Colanic acid (M-antigen)	-	-	+	+	+	+

^a ^*ssrS *is annotated as an rRNA in Crooks but in K-12 and W it is annotated as an ncRNA. It is included in this table as an ncRNA

The total number of genes, tRNA, other ncRNAs and IS elements in each strain includes pseudogenes/pseudo-tRNAs etc.; the number of pseudo-elements in each case is in noted in brackets. A '+' means the element is present; a '-' means the element is absent. ND = not determined in annotation. Safe laboratory strains: W (ATCC 9637) and its plasmids pRK1 and pRK2; K-12 (MG1655); B (REL606); and Crooks (ATCC 8739). W is in phylogroup B1; K-12, B and Crooks are in phylogroup A.

A wide variety of algorithms were used to predict and annotate coding and non-coding genes (see Methods). Like the three other sequenced laboratory strains, W has 22 rRNA genes expressed from 7 rRNA operons; these operons are present at similar locations in all four genomes. The four strains share 85 tRNAs and there are four unshared tRNAs located in large mobile elements. W has *thrX *and *tyrX*, which occur within a variable region of the Rac*W prophage and are homologous to *thrU *and *tyrU *of *E. coli *K-12; due to separate IS-mediated deletions, W and B are both missing a tRNA which occurs upstream of *ypjC *in K-12; in K-12, *ileY *is present. In Crooks the sequence of a tRNA in the same location is identical to *ileY *of K-12 but has been mis-annotated as a tRNA-Met2 variant.

**Figure 2:**
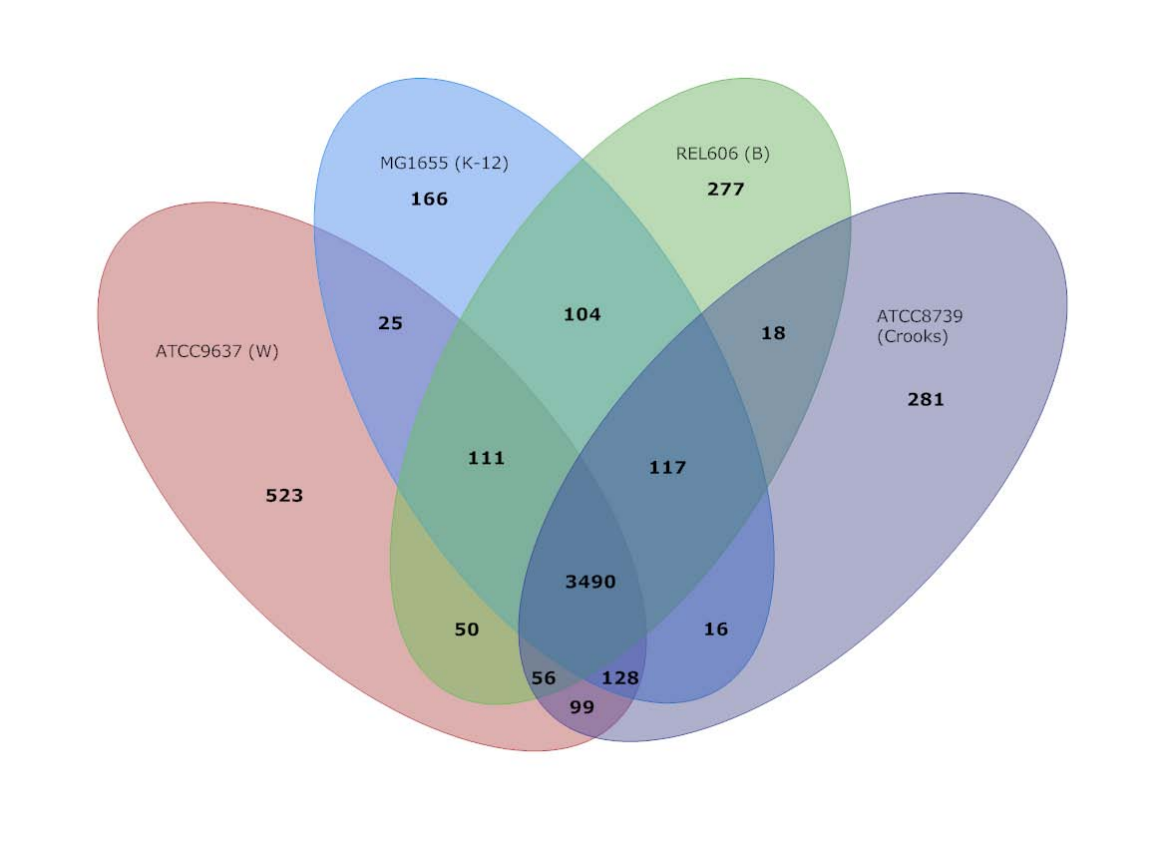
**Comparison of orthologous CDSs between W, K-12, B and Crooks strains**. The number of shared genes, as well and the number of unique genes and genes shared between one, two, and three strains are shown. All-against-All BLASTP for amino acids (E-value ≤ 1E-5, identity ≥ 90%, coverage ≥ 80%) was used to assign orthologs. Total CDS counts for K-12, B & Crooks differ by 8, 14 & 5 respectively as some CDSs had more than one ortholog in another genome (Additional File 1).

All-against-all BLASTP comparison of chromosomal protein-coding orthologs among the four safe laboratory strains (Figure 2, Additional File 1) showed that of 4,482 predicted CDSs in W, 3,490 are shared among these four strains. Another 413 are found in at least one other strain, leaving 523 CDSs that are unique to W. Consistent with the larger genome size, this is ~320-360 more CDSs than were found to be unique in any other safe strain. It should be noted that the number of shared orthologs between strains is not an indicator of overall relatedness, since increases in shared genes tends to arise from large insertion elements (for example, K-12 and B share a large genomic island encoding a restriction modification system while Crooks and W share two large gene clusters encoding excretion systems). Furthermore, differences in genome sizes bias this kind of relationship comparison.

**Figure 3:**
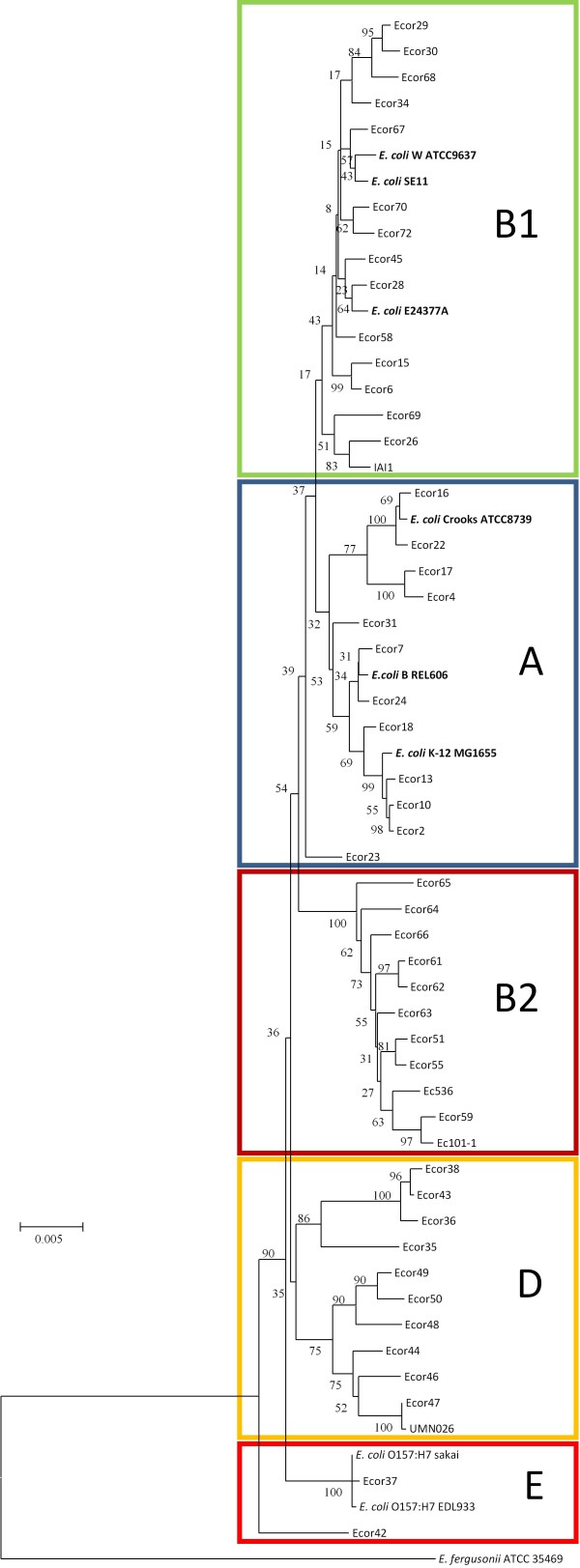
**Phylogenetic analysis of sequenced E. coli strains**. Phylogenetic relationships based on seven housekeeping genes (adk, fumC, gyrB, icd, mdh, purA, and recA). Strains cluster into phylogroups; W can be found in group B1, whereas the other three laboratory strains are in group A. Escherichia fergusonii (ATCC 35469) was used as an out-group. The tree shows bootstrap values (percentage per 1000 replicates). The scale bar represents divergence time.

*E. coli *strains can be divided into five different ECOR phylogroups (A, B1, B2, D and E) based on the sequences of housekeeping genes [34]. Commensal strains are found primarily in group A or group B1, which are sister groups, while pathogenic strains are generally found in Group B2, D and E [31,34,35]. A phylogenetic tree was constructed by sequence concatenation of seven housekeeping genes [36] (Figure 3). Using this approach, W was assigned to group B1. Group B1 contains a large number of commensal strains [37]. The other three sequenced safe strains (K-12, B and Crooks), are all members of phylogroup A [31,35]. Interestingly, these groupings are consistent with genome sizes of sequenced strains: group B1 strains have larger genomes than group A strains. W is arguably a more appropriate strain than K-12, B or Crooks for comparison with commensal and pathogenic strains of phylogroup B1.

### Plasmids

An early report suggested that *E. coli *W contains three plasmids [38]. However, it was later suggested that W contains only two plasmids [26]. Our sequence data confirmed the latter report: W contains two plasmids, pRK1 and pRK2. pRK1 is a circular plasmid of 102,536 bp. It encodes 118 genes: 114 protein coding genes, one pseudogene and three ncRNAs (Table 1). BLAST analysis demonstrated that it belongs to Incompatibility Group I1 (IncI1) and has high structural similarity with the IncI plasmids pR64 (a reference IncI1 plasmid), pSE11-1 (a plasmid of roughly 100 Kbp isolated from *E. coli *SE11), and pColIb-P9. Analysis of *inc*, a marker for IncI designations [39], showed that *inc *in pRK1 differed by only one base pair from the reference *inc *of Inc I1 subgroup Iγ [40]. IncI1 plasmids are characterized by the presence of genes encoding a thick pilus, a thin type IVB pilus, the pilus-associated protein gene *pilV*, and the DNA primase gene *sog *[41].

Genes for antibiotic resistance are found on most sequenced IncI plasmids, including IncI1 plasmids [42] and IncIγ-type R621a [43]; however, pRK1 does not encode any antibiotic resistance genes. This is desirable in industrial strains as genetic manipulation for strain improvement often involves the use of antibiotic selection. In addition, an IS*91 *insertion has interrupted two genes involved in colicin production (*cib *and *imm*). This insertion also resulted in the introduction of genes involved in κ-type fimbriae (see further comments below).

The *trbA*-*exc *region in IncI1 plasmids is a diverse region and includes genes that are involved in plasmid maintenance and transfer. pRK1 contains a complete *trb *regulon, which is required for plasmid transfer. Two other genes are of interest: *excAB*, which controls surface exclusion and thus determines which plasmid types can conjugate into the host cell, and *pndCA*, which controls plasmid stability [44]. In pRK1, *pndCA *has been lost, suggesting that plasmid stability might be affected even though there is no direct evidence that pRK1 is unstable in W. In addition, the 3' region of *exc *differs greatly from other *exc *genes on IncI1 plasmids, suggesting that this gene encodes a protein which determines different mating specificity than other IncI plasmids.

Plasmid pRK2 has been sequenced previously [45] and our analysis is in agreement with the reported information. Briefly, pRK2 is a cryptic ColE1-type plasmid; it is 5,360 bp and encodes 16 predicted genes including 15 protein-coding genes and one non-coding RNA. It is stably inherited and contains four putative mobilisation genes and a gene encoding a Rom protein. It shares 99% identity with pSE11-4, a plasmid isolated from the group B1 commensal *E. coli *SE11 [31].

Finally, there is some evidence that *E. coli *W once harbored a third plasmid. An IS*91 *insertion in pRK1 (see below for further details) is homologous to a region in pSE11-3, an IncF plasmid from *E. coli *SE11 [31]. The insertion has deleted a region of pRK1 which is normally found in IncI plasmids. Additionally, the partial fimbrial gene cluster which was transferred with the insertion is known to be plasmid-encoded [46]. W and SE11 belong to the same phylogroup and therefore might share a common ancestry; furthermore, two of the SE11 plasmids are highly similar to pRK1 and pRK2 (pSE11-1 and pSE11-4, respectively). Thus, it seems likely that an ancestral W strain might have harbored a plasmid similar to pSE11-3.

### Mobility elements and defence systems

*E. coli *genomes consist of a conserved core interspersed with variable regions encoding accessory functions [47]. The conserved core is shared with closely related genera such as *Citrobacter *[48], *Shigella *[49] and *Salmonella *[50]. The accessory genome encodes lifestyle-specific functions which are often found in large clusters or related genes (so called 'genomic islands') [51-53]. These clusters contain a different G+C content compared to the rest of the genome (see Figure 1) and are acquired through horizontal gene transfer (HGT) *via *natural transformation, bacteriophage-mediated transduction or conjugation.

#### Mobility elements

Large genomic islands which are flanked by mobility elements are known as large mobile elements (LMEs), and include prophages or phage-like elements (pLEs) [54]. Differentiation between prophages and pLEs can be difficult; in general, a prophage will contain specific metabolic and structural genes associated with a prophage, while a pLE will contain an integrase and very few regions which are homologous to known prophages. LMEs carry large complements of genes which might confer a variety of metabolic attributes. *E. coli *W has six prophages and three pLEs, the latter of which we have designated '*E. coli *W phage Like Elements' (WpLEs). A detailed list of LMEs in *E. coli *W and other safe strains can be found in Table 2.

**Table 2:** Large mobile elements found in safe strains.

Insertion site	W	K-12	B	Crooks
*c *- *mom*	WMu (Mu)	-	-	-
*thrW *tRNA	-	CP4-6 (CP4)	-	-
*argU *tRNA	-	DLP12 (λ)	DLP12 (λ)	-
*ybhC-ybhB*	-	-	λ*B	λ*Cr
*rybB *ncRNA	Rybb*W (P2)	-	Rybb*B (P2)	Rybb*Cr (P2)
*icdA*	-	e14 (λ)	-	-
*ompW*	-	-	-	-
*ttcA*	Rac*W (λ)	Rac (λ)	Rac (λ)	-
*ydfJ*	Qin (λ)	Qin (λ)	Qin (λ)	Qin (λ)
*cobU*-yeeX	-	CP4-44 (CP4)	CP4-44 (CP4)	-
*cyaR *RNA	Wphi2 (P2)	ogr-D'	P2*B	-
*argW *tRNA	Argw*W (λ)	CPS-53 (KpLE1)	-	-
*eutA*	-	CPZ-55 (CP4)	-	CrpLE1
*ssrA *tmRNA	WpLE1	CP4-57 (CP4)	Ssra*B^a^	CrpLE2
*pheV *tRNA	-	-	Phev*B (CP4)	CrpLE3
*selC *tRNA	WpLE2	-	Selc*B (CP4)	Selc*Cr (CP4)
*pheU *tRNA	-	-	-	Pheu1*Cr (CP4)
*cpxP-fieF*	Wphi1 (P2)	-	-	-
*pheU *tRNA	-	-	-	Pheu2*Cr(CP4)
*leuX *tRNA	WpLE3	KpLE2	KpLE2	KpLE2^b^

^a ^Prophage type is unknown

^b^ KpLE2 P4 integrase is interrupted by IS3

A '-' means that no mobile element was found at that insertion site. Prophage types are shown in brackets. Strains are W (ATCC 9637), K-12 (MG1655), B (REL606), Crooks (ATCC 8739).

A total of twenty-eight LMEs are annotated amongst the safe *E. coli *strains. They are spread out over nineteen different sites in the chromosome and all but one can be classified as either a pLE or one of three different prophages (P2-like, P4-like or λ-like). The exception is the Mu prophage, a transpositional phage that inserts into almost random chromosomal locations [55]; among the four strains, Mu prophage is only found in W. None of the LMEs in W encode any genes of particular note. In the other strains, a few genes of interest are encoded on prophages. Rybb*B carries retron Ec86 [6], which encodes a reverse transcriptase that is missing from Rybb*C and Rybb*W. The P4 prophage CP4-44 is absent in W and Crooks but present in K-12; the *flu *gene is encoded on this prophage in K-12 and is encoded on Phev*B in B. The λ prophage is the most promiscuous prophage element among the four strains.

Ten pLEs are found among safe strains. Only KpLE2 is shared (being found in both K-12 and B). *E. coli *Crooks might have harboured KpLE2: it contains a 259 bp pseudogene, the first 137 bp of which shares 72% identity with the P4-integrases of KpLE2 in K-12 and B. KpLE2 contains the *fec *regulon (discussed below) and the *sgc *operon, which is involved in pentose and pentitol sugar breakdown [56]. K-12 contains KpLE1, which includes the *gtrAB *regulon encoding a bactoprenol glucosyl-transferase involved in O-antigen modification. The Crooks strain harbours CpLE1, which contains an endonuclease, and CpLE3 which also contains a *fec *regulon. The WpLE3 of W appears to comprise two separate pLEs, as a second P4-integrase is found with distinct regions of DNA following each integrase. The first region contains a toxin-antitoxin system while the second region contains a putative 5-methylcytosine restriction system.

Insertion sequences (ISs) play an important role in the cell's ability to evolve and adapt to new environments [57]. A complete description of the IS elements in safe strains can be found in Table 3. Only two ISs are conserved among all four strains; as previously reported [58], no copies of IS*1 *were found within the W genome. The W genome contains 24 IS elements, which is significantly fewer than K-12, B or Crooks; as a consequence, W has no IS-related gene inactivation occurring in the chromosome, whereas K-12 and B both have a number of genes inactivated. These include genes involved in lipopolysaccharide (LPS) and capsular polysaccharide (CPS) synthesis, as well as large deletions such as the 41 Kbp region between *uvrY *and *hchA *in B which removes the Flag-1 flagella-encoding gene cluster (see below for further details).

**Table 3:** Insertion sequences found in safe strains.

IS	Gene	W	K-12	B	Crooks
IS*1*	*insAB*	0 (0)	7 (0)	28 (0)	19 (0)^a^
IS*1*H	*insXY*	1 (0)	0 (0)	0 (0)	0 (1)
IS*2*	*insCD*	0 (0)	6 (1)	0 (2)	0 (0)
IS*3*	*insEF*	3 (0)	5 (2)	5 (2)	1 (0)
IS*4*	*insG*	0 (0)	1 (0)	1 (0)	0 (0)
IS*5*	*insH*	0 (0)	11 (0)	0 (0)	2 (0)
IS*30*	*insI*	0 (0)	3 (1)	0 (1)	4 (0)
IS*91*		1 (0)^b^	0 (0)	0 (0)	0 (0)
IS*150*	*insJ*	2 (0)^b^	1 (0)	4 (1)	0 (0)
IS*186*	*insL*	0 (0)	3 (0)	5 (0)	3 (0)
IS*600*		0 (0)	0 (1)	1 (0)	0 (0)
IS*609*	*tnpAB*	4 (0)	1 (0)^c^	1 (0)	0 (2)
IS*621*		4 (1)	0 (0)	0 (0)	0 (0)
IS*911*	*insO*	2 (0)	0 (3)	1 (2)	0 (0)
ISEcB*1*		0 (0)	0 (0)	1 (0)	0 (0)
ISEhe*3*	*insX*	0 (0)	0 (1)^d^	0 (1)	0 (1)
ISEc*14*		0 (0)	0 (0)	0 (0)	3 (0)
ISEc*17*		0 (0)	0 (0)	0 (0)	3 (0)
ISZ'	*insZ*	0 (0)	1 (0)	0 (0)	0 (0)
ISSd*1*		0 (0)	0 (0)	0 (0)	0 (2)
Total		16 (1)	38 (9)	47 (9)	35 (6)

^a ^Includes IS*1 *family elements

^b ^Found on plasmid pRK1

^c ^Annotated as predicted transposase in K-12 (MG1655) genome (locusTag b1432). Predicted to be IS*609 *by ISFinder

^d ^Annotated as ISX in K-12 (MG1655) genome. Predicted to be ISEhe*3 *by ISFinder

Genes encoded on insertion sequences are noted; the number of additional pseudogenes is noted in brackets. Strains are W (ATCC 9637), K-12 (MG1655), B (REL606), Crooks (ATCC 8739).

#### Restriction modification and CRISPR systems

Restriction modification and clustered regularly interspaced short palindromic repeat (CRISPR) systems play an important role in antiviral defence against invasive foreign genetic material (*e.g.*, bacteriophages and integrative elements) and hence control the extent of HGT [59]. Restriction capabilities are conferred by the immigration control region [60]. Both W and Crooks are restriction minus as they lack *hsdMRS, mcrBC and mrr*, which encode the restriction modification complexes. In W, this cluster has been replaced by the *pac *gene encoding a penicillin *G acyclase* (PGA), which catalyses the breakdown of penicillin G into phenylacetic acid and 6-aminopenicillanic acid [17]. This capability has been exploited for the industrial production of PGA using *E. coli *W [16]. In Crooks, the immigration control region has undergone multiple changes due to IS element insertions. The lack of restriction modification systems in W and Crooks suggests that these strains are less able to inactivate foreign DNA.

CRISPR systems inhibit horizontal gene transfer. The detailed mechanisms have just begun to be exposed [61]. Recently, two CRISPR systems have been described in *E. coli*: CRISPR2 and CRISPR4 [62]. These systems differ by the presence or absence of CRISPR associated sequence (CAS) proteins (the function of which is unknown), and by the location, number and sequence of repeats. *E. coli *W contains three CRISPR2 arrays, CRISPR2.1, 2.2, and 2.3 (Table 4). Genes encoding *E. coli *Cas proteins are present next to CRISPR2.1. W also contains the CRISPR4.1-2 array but not the associated *Yersinia pestis *Cas proteins, which are found in many *E. coli *strains [62]. Each safe strain has the same number of arrays, but the sequences and number of repeat regions varies (Table 4). There are two *cas *gene clusters found in *E. coli *which vary in the *cas3*-*cse3 *region; it is unclear if they have the same function [63]. One is found in K-12 and Crooks and the other is found in W and O157. Multiple insertions and deletions have destroyed the *cas *gene cluster in *E. coli *B.

**Table 4:** CRISPR arrays found in safe strains.

	CRISPR array			
	2.1	2.2	2.3	4.1-2
W	16^a^	3	11	2
K-12	14^a^	3	7	2
B	5	3	14	2
Crooks	22^a^	3	29^b^	4

^a ^CAS-E genes proceed array

^b ^IS element occurs within array

Strains are W (ATCC 9637), K-12 (MG1655), B (REL606), and Crooks (ATCC 8739).

### Virulence/Fitness Factors

Virulence factors are classically considered to be associated with host interactions and pathogenicity. However, it should be noted that many of these so-called virulence factors can also be considered fitness factors in a non-virulence context [64]. For example, adhesins are important for colonizing all manner of niches; colonisation does not necessarily lead to infection and disease.

#### Serotypic antigens

*E. coli *serotypes are defined according to the polysaccharide component of LPS molecules [65-67]. These include CPSs, which can be either K-antigen or colonic acid (M-antigen) and O-polysaccharides (O-antigen). The H-antigen is used for serotyping, and its type is usually determined by FliC, a flagellar structural protein [68]. HGT of the gene regions responsible for production of O-antigen, K-antigen, H-antigen, and the LPS core has lead to a high degree of variability [69]. There are 167 different O-antigen types and 80 K-antigen types currently recorded amongst *E. coli*. Whereas other safe *E. coli *strains have accumulated IS-mediated deletions in antigenic clusters (Table 1), W has intact clusters. It has an R1 type LPS core and an O6 type O-antigen. Type O6 is widely distributed and found both in uropathogenic *E. coli *(UPEC) strains and in commensal strains [70]. W does not produce a K-antigen, but it has the gene cluster involved in colonic acid synthesis; colonic acid resembles K-antigen group IA capsular polysaccharides [66]. It also has the phosphorelay regulon (encoded by *rcsA *and *rcsDBC*) which activates production of colonic acid. FliC homology suggests that *E. coli *W produces an H49 type H-antigen [71]. W can thus be antigenically characterised as *E. coli *W (O6:K-:H49) CA^+^.

#### Adhesins

Fimbriae and other adhesins determine whether *E. coli *can bind to and colonise specific environments, including different types of cells. They are associated with virulence in pathogenic strains of *E. coli *such as enteroaggregative *E. coli *55989 (EAEC) [72] but are also key to the fitness of probiotic *E. coli *strains such as strain Nissle 1917, as they allow it to colonize the human intestine [73]. In W, there are thirteen chromosomal gene clusters involved in fimbrial biosynthesis, and most of these are conserved among the safe strains of *E. coli *(Table 5). Differences arise in genes encoding the fimbrial usher protein and the tip adhesins. Tip adhesins are important determinants of host cell specificity during pathogenesis; the usher protein is a membrane protein which is involved in the assembly of a fimbria and determines which group the fimbria belongs to [74].

**Table 5:** Fimbrial gene clusters found in safe strains and in representative Group B1 strains.

Insertion site (W)	**Type**^**a**^	W	K-12	B	Crooks
Chromosome					
*yadN-ecpD-htrE-yadMLKC*	γ_4_	+	+	+	*ECs0145-ECs0139*^b^
*ecpABC-yagW-ecpE*	α	+	+	+	+
*sfmACDHF*	γ_1_	+	+	+	+
*ybgDQPO*	π	+	+	+	+
*elfADCG-ycbUVF*	γ_1_	+	+	+	+
*csgDEFG-csgBAC*	curli	+	+	+	+
*egoABCDEF*	γ_1_	+	*ΔegoABC*	*ΔegoAB*	*ΔegoAB*
*yehDCBA*	γ_4_	+	+	-	+
*esoABCDEFGH*	π	+	*yfcOPQRSTUV*	*yfcOPQRSTUV*	*yfcOPQRSTUV*
*ygiL-yqiGHI*	π	+	IS*2::yqiG*	+	+
*eafABCD*	α	+	-	-	+
*yraHIJK*	γ_1_	+	+	+	+
*gltF-yhcF^c^*	β	-	IS*5::yhcE*	-	-
*lpfABCDE*	γ_1_	+	-	-	-
*lpfA2-D2*	γ_1_	+	-	-	-
*fimAICDFGH*	γ_1_	+	+	+	IS3::fimG, *fimAICDF

**Plasmids**					

*faeCDEFGH*	κ	*ΔfaeHIJ*^d^	-	-	-

^a ^Type based on [74]

^b ^Crooks contains a related γ_4 _fimbrial found in *E. coli *O157:H7 at this location

^c ^Cluster location in *E. coli *K-12 MG1655

^d ^Cluster located on W pRK1

A '+'means the element is present; a '-' means the element is absent. Where some genes from the cluster are deleted, this is noted as e.g. *egoABC*. If a different gene fimbria gene cluster is present in the insertion site, the alternative gene cluster is noted. Safe laboratory strains are W (ATCC 9637), K-12 (MG1655), B (REL606), and Crooks (ATCC 8739). W is in phylogroup B1; K-12, B, and Crooks are in phylogroup A.

There are 2 α-type fimbrial gene clusters in W: *ecpABC-yagW-ecpE*, and a novel fimbrial gene cluster found between *exuT *and *exuR*. We have designated this novel cluster *E. coli *α-type fimbria, *eafABCD*. However, neither of the clusters in W contains a gene encoding for the tip adhesin protein, which is found in other α-type fimbrial clusters and is responsible for cell binding [75]. Thus, it is unlikely that the W α-type fimbriae can function in pathogenesis or colonisation of cells in general.

W contains five γ_1_-type fimbrial gene clusters. One of these is *E. coli *YcbQ laminin-binding fimbria (ELF, formerly *ycbQRST*) [76] which is shared between group B1 strains. In W, the major subunit protein ElfA is relatively different (84% identity) from that found in K-12 and O157:H7 EDL933. Deletion of this gene in O157:H7 EDL933 has been shown to lead to a significant reduction in ability to adhere to HEK293 cells [76]. A γ_1_-type cluster found in *E. coli *O157:H7 and annotated as *ECs2113-ECs2107*, is also present in W. This cluster is also present in *E. coli *K-12 (annotated as *ydeQRST*), but a deletion removes *ECs2113-ECs2112 *and truncates *ECs2111 *(which normally encodes the usher protein). We have designated this gene cluster *E. coli *γ-type 1, with the operon consequently designated *egoABCDEF*. Information on the other three γ_1_-type fimbrial gene clusters is limited but all are found in K-12 and are cryptic or poorly expressed under classic laboratory conditions [77].

Two groups of fimbriae closely related to γ_1_-type fimbriae and known as long polar fimbriae [78] are also found in *E. coli *W. They are commonly found in both pathogenic and commensal strains of *E. coli *and consist of 3-6 genes. The first cluster, *lpfA1-E1*, is found in other *E. coli *group B1 strains (Table 5) and shows 44-77% amino acid identity to the *lpf *gene cluster of *Salmonella enterica*. The adherence of *lpfA1-E1 *homologs in other *E. coli *strains is known to vary depending on both the sequence of the gene cluster and on its regulation [78-80]. The second cluster, *lpfA2-D2*, is identical to the *lpf *operon found in *E. coli *789. This *lpf *operon has been shown to produce the fimbria responsible for adherence to human HEK293 cells [81].

There are also three π-type fimbrial gene clusters in W and the other safe strains. One of these, located between *sixA-yfcN *and consisting of seven genes, shows >95% sequence identity with a fimbrial gene cluster located in the same chromosomal position in O157:H7. In O157:H7, this cluster is annotated as *ECs3222*-*ECs3216*; we have designated it *E. coli *π-type one, with the operon consequently designated *epoA-H*.

Due to an insertion event on pRK1, W has five of the eight genes from the κ-type *csh *fimbrial gene cluster. However, the lack of the terminal three genes most likely renders this cluster non-functional.

Antigen-43 is a protein which works synergistically with fimbriae to promote adhesion [82]. It is encoded by the *flu *gene on the prophage CP4-44 [77], which is present in *E. coli *K-12 and B, but is absent in W; consequently, antigen-43 is also absent in W.

Pili are involved in gene transfer and thus in obtaining pathogenicity factors and other elements. They also affect biofilm formation, which is an important consideration for industrial fermentation. Plasmid pRK1 contains the 14-gene *pil *cluster which encodes a type IVB thin pilus involved in liquid mating [83]. In contrast to R64 and ColIb-P9, pRK1 does not contain the recombinase gene *rci *or repeat-flanked shufflon regions that increase the host adhesion variability of the thin pilus [84]. In addition, there are mutations in *pilS *and *pilU*, which encode essential functions for pilus activity. The resulting PilS protein has three amino acid mutations at positions where mutations have been shown to limit or inactivate pilus function [85]. PilU has three amino acid mutations at positions which severely affect transfer frequency [86]. Furthermore, the PilS and PilU proteins have an additional 33 and 12 amino acid changes, respectively, at positions which have not been previously characterised. Additionally, *E. coli *C producing the PilVA-type thin pilus forms cell aggregates in liquid culture due to the pilus activity [87], whereas *E. coli *W does not (data not shown). All of these considerations suggest that *E. coli *W does not form thin pili.

Plasmid pRK1 also contains a set of transfer genes, comprising 29 genes over 3 operons, which encode a thick pilus involved in both surface and liquid mating [88]. The pRK1 complement includes all but one of the *tra *genes: the *traABCD *operon is incomplete as it is missing *traD*, a non-essential thick pilus protein of unknown function [89].

#### Secretion Systems

Secretion systems are required for the transport of proteins across the cell membrane and play a role in virulence [90] and fitness [91]. The conservation of core genes between flagellar systems and Type III secretion systems has led some authors to recognise the flagellar export mechanism as a type of secretion system [92]. Consequently, there are seven secretion systems in *E. coli *[90].

Flagella are required for cellular propulsion. There are two flagella systems in *E. coli *[93]. In addition to the well known Flag-1 flagellar cluster common in *E. coli*, W has a Flag-2 gene cluster. The Flag-2 locus has been found in many genera of gammaproteobacteria, including *Vibrio parahaemolyticus *[94], *Escherichia coli *[93], *Yersinia enterolitica *[95], *Citrobacter rodentium *[48] and *Aeromonas hydrophila *[96]. The *V. parahaemolyticus *and *A. hydrophilia *Flag-2 systems have been shown to be active experimentally [94,96]. In *E. coli*, it is found in some strains but not others; it was originally assigned in *E. coli *042 by homology [93] but has never been shown experimentally to be functional. In *E. coli *042, *lfgC *(*flgC *in other genera), which encodes a rod protein required for protein export through the outer membrane, has a frameshift mutation, suggesting that the Flag-2 system is not functional. In support of this, a swarming motility assay was negative [97]. *E. coli *W and Crooks both contain a Flag-2 locus. The *lfgC *genes are not mutated, but a two-gene toxin/anti-toxin system found in 042 between *lafW *and *lafZ *is absent. Both strains are missing *motY*, which encodes a motor protein essential for swarming in *V. parahaemolyticus*; in addition, they do not contain *maf-5*, a modification accessory factor essential for a functional lateral flagellar in *A. hydrophilia *[96]. W (but not Crooks) contains a Mu prophage located in a non-coding region of the Flag-2 locus (between *EcolC_3376 *and *EcolC_3377*). Together, these observations suggest that the Flag-2 locus is not functional in *E. coli *W or in Crooks. In K-12 and B, all that is left of the Flag-2 system are the two terminal remnants, *fhiA *(*lfhA *pseudogene) and *mbhA *(*lafU *pseudogene) [93].

**Figure 4:**
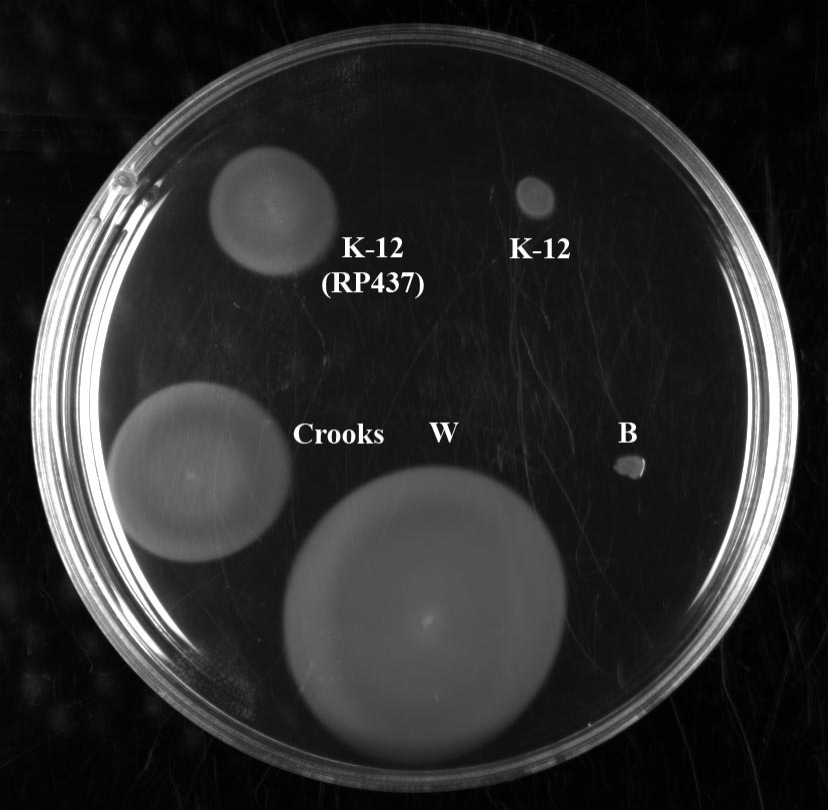
**Swarming motility assay**. A swarming motility assay was performed using E. coli strains W, Crooks, K-12 (MG1655), K-12 (RP437), and B. B was negative; K-12 (MG1655) showed very minimal swarming, while K-12 (RP437), Crooks and W were positive. Assays were performed in triplicate at 25°C and at 37°C; results were similar at both temperatures (figure shows representative results from 25°C incubation).

A swarming motility assay was performed to examine functionality of the Flag-2 locus (Figure 4). Consistent with loss of the Flag-2 locus, *E. coli *B does not swarm. However, despite the loss of what appear to be essential Flag-2 genes, W and Cooks strains both swarm. Although the swarming assay has been used to assess Flag-2 activity [93,96], it should be stressed that the test is not specific to Flag-2. *E. coli *K-12, which has clearly lost the Flag-2 locus, shows very limited swarming; however a K-12 mutant (RP437) exhibits a swarming phenotype even though it does not contain a Flag-2 locus [98]. Further analysis by specific deletion will be required to determine whether or not the Flag-2 locus is active in W.

There are two Type II secretion systems (T2SSs) in *E. coli*. T2SSs are required for toxin export from cells [99] as well as a variety of other proteins which affect fitness for specific environments [64]. *E. coli *K-12, B, and Crooks all carry a repressed 14-gene T2SS gene cluster (*gspA-O*, located between *rpsJ *and *bfr*) [100]. This T2SS has been lost in W due to a *gspO-rpsJ *deletion. Both W and B (but not K-12 or Crooks) carry the second T2SS gene cluster (*yghJ-pppA-yghG-gspC-M*). Unlike *E. coli *B, in which *gspL *is truncated, all genes in W appear functional. However, it should be noted that unlike K-12, which can export chitinase through an expressed T2SS [100], the W genome does not contain any known genes encoding enzymes or toxins that can be exported through T2SSs.

Type III secretion systems (T3SSs) inject effector proteins into recipient cells leading to pathogenic or pro-survival responses [101]. There are two T3SSs in *E. coli*: the *E. coli *Type III secretion systems 1 and 2 (ETT1 and 2) [102]. ETT1 is absent in all four sequenced laboratory strains. Remnants of the ETT2 locus can be found in all of them, but they do not have a functional ETT2. Mutational attrition of ETT2 is common in *E. coli *strains [103].

Type VI secretion system (T6SS) gene clusters consist of 15 to 25 genes and have been identified in numerous Gram-negative Proteobacteria [104]. In some T6SSs, the genes encoding the secreted proteins, Vgr and Hcp, are found in different locations of the genome [105], but commonly next to *rhs *genes [106]. This is the case in W, which contains two T6SSs. The structure of the first gene cluster is homologous to the system previously described in *E. coli *O157:H7 Sakai [107]. It consists of 17 genes and is termed the 'enterohaemorrhagic *E. coli *type six secretion system cluster' (EHS) [48]. However, this system is found in numerous other non-pathogenic strains, including SE11 and HS (data not shown). A second T6SS is located downstream of *metV *and is homologous to the T6SS found in *E. coli *CFT073 [108], also located downstream of *metV*. We have designated this cluster *Escherichia coli *type six secretion system cluster 2 (ETSS2) as the EHS is cluster 1. In W, it is most likely deactivated due to an IS621-mediated insertion. W is the only safe strain which contains a T6SS, although none of the effector molecules which are transported into host cells [104] are present. Therefore, this system is unlikely to function in pathogenicity.

#### Rearrangement hot spot (Rhs) elements

Rhs elements are large highly repetitive regions; they constitute roughly 1% of the *E. coli *genome [109]. They are composed of four elements: a clade-specific N-terminal domain, a core domain, a hyperconserved domain, and a variable C-terminal domain [106]. Often, partial core domain and variable C-termini regions (called C-terminal tips) are observed downstream of intact *rhs *genes. These are proposed to play a role in intra-rhs variability [106]. C-terminal tips have occasionally been annotated as insertion sequences in the ISFinder database due to the presence of an H-repeat (H-rpt), although transposition activity has not been observed [110]. *E. coli *W contains seven *rhs *genes (*rhs1-rhs7*; Table 6), two of which are deactivated due to frame-shift mutations. Of the remaining five, four have downstream C-terminal tips of varying number. Both Crooks and W also possess type IV Rhs elements; these are missing in K-12 and B.

**Table 6:** Rearrangement hot spot (Rhs) elements found in safe strains.

RHS	Region (K-12)	W	K-12	B	Crooks
1	b0215-b0221	*rhsW1 *(0)	0 (0)	0 (0)	0 (0)
2	b0496-b0503	*rhsW2*^a ^(1)	*rhsD *(1)	*rhsD *(1)	0 (0)
3	b0570-b0569	*rhsW3 *(3)	0 (0)	0 (0)	*EcolC_3079 (3)*
4	b0699-b0706	*rhsW4*^a ^(1)	*rhsC *(1)	*rhsC *(1)	*EcolC_2955 (0)*
5	b1455-b1461	*rhsW5 *(0)	*rhsE*^a ^(0)	*rhsE *(0)	*EcolC_2201 (0)*
6	b1976-b4497	0 (0)	0 (0)	0 (0)	*EcolC_1663 (0)*
7	b1988-b1990	0 (0)	0 (0)	0 (0)	*EcolC_1653 (0)*
8	b3481-b3485	0 (0)	*rhsB *(0)	*rhsB *(0)	*EcolC_0234 (0)*
9	b3592-b3596	*rhsW6 *(1)	*rhsA *(1)	*rhsA *(1)	*EcolC_0120 (1)*
10	b3936-b3937	*rhsW7 *(0)	0 (0)	0 (0)	*EcolC_4081 (0)*

^a ^*rhs *gene is a pseudogene

Positions are based on K-12 annotation (U00096). The number of C-terminal tips is shown in brackets. Strains are W (ATCC 9637), K-12 (MG1655), B (REL606), and Crooks (ATCC 8739).

### Comparison with other group B1 strains

We performed a comparison between W and other sequenced group B1 strains, including the commensal strains SE11 and IAI1, and a variety of pathogenic strains: EAEC strain 55989, ETEC strain E24377A, and EHEC strains O26, O103, and O111 (Table 7). The chromosome size is relatively variable, ranging from 4.7 Mbp (IAI1) to 5.7 Mbp (O26). A backbone genome can be defined for each strain by subtracting the LMEs (including plasmids and integrative elements) from the total genome size (Table 7). Interestingly, the size of this backbone genome is very similar (ca. 4.5 Mbp +/- 83 Kbp) for all strains. The backbone sequences are not identical; differences are found primarily in the presence or absence of large structural elements encoding secretion systems (including flagella) and adhesins. For example, the Flag-2 is found W and the two EHEC strains O26 and O111 (but not in the EHEC strain O103 or in other pathogenic strains, or in the commensal strains) (Table 8). W has the largest backbone genome (4.588 Mbp) as it has the largest number of large structural elements (T2SS, T3SS, T6SS and flagella). No group B1 strain contained the T2SS *gspA-gspO *which is present in group A. *E. coli*. W contains the smallest number of insertion sequences of all B1 strains; these sequences also play a role in attrition, since recombination between them may result in loss of large regions of DNA [111]. Additionally, each of the group B1 strains examined contains the *csc *regulon for permease-mediated sucrose utilisation.

**Table 7:** Comparison between sequenced Group B1 strain genome features.

	Safe	Commensal		EAEC	ETEC	EHEC		
	
Strains	W	SE11	IAI1	55989	E24377A	O26	O103	O111
**Version**	CP002185.1	AP009240.1	CU928160.2	CU928145.2	CP000800.1	AP010953.1	AP010958.1	AP010960.1
**Chromosome size (Mbp)**	4.901	4.888	4.701	5.155	4.980	5.697	5.449	5.371
**CDSs**	4482	4679^a^	4356	4766	4634	5368	5058	4976
**Large Mobile Elements**	12	16	5	14	22	34	23	30
**Prophage regions**	7	7	3	5	8	19	15	17
**Integrative elements**	3	3	2	8	7	11	7	8
**Plasmids**	2	6	0	1	7	4	1	5
**Total IS Elements**	18 (6)	33 (ND)	42 (ND)	150 (ND)	80 (ND)	135 (ND)	116 (ND)	119 (ND)
**Genome Backbone Size (Mbp)**	4.588	4.511	4.529819	4.504999	4.536845	4.564564	4.520522	4.536492
**Total Mobile Element Size**	0.421363	0.644488	0.171181	0.722345	0.810839	1.290967	1.004338	1.229646
**Total genome size (Mbp)^b^**	5.009	5.156	4.701	5.227	5.348	5.856	5.525	5.766

^a ^- pseudogenes were not calculated in the SE11 genome paper

^b ^- includes size of plasmids

The total number of genes, tRNA, other ncRNAs and IS elements in each strain includes pseudogenes/pseudo-tRNAs etc.; the number of pseudo-elements in each case is noted in brackets. Note that ncRNAs are not annotated/incompletely annotated in SE11 and E24377A, respectively; conseqeunctly, the absolute number of genes shown for these strains is inaccurate. ND = not determined in annotation.

**Table 8:** Large structural components found in Group B1 strains.

Strain	Flag-1	Flag-2	T2SS	ETT1	ETT2^a^	EHS	ETSS2
**W**	**x**	**x**	**x**	**-**	**x**	**x**	**x**
**SE11**	**x**	**-**	**-**	**-**	**x**	**x**	**-**
**IAI1**	**x**	**-**	**-**	**-**	**x**	**x**	**-**
**55989**	**x**	**-**	**-**	**-**	**x**	**x**	**-**
**E24377A**	**x**		**-**	**-**	**x**	**x**	**x**
**O26**	**x**	**x**	**x**	**x**	**x**	***ΔetsH-etsG***	**-**
**O103**	**x**	**-**	**x**	**x**	**x**	**x**	**x**
**O111**	x	x	-	x	x	x	-

^a ^- This locus is inactive in each group B1 strain

Presence (x) or absence (-) of large structural elements in group B1 strains. Flag-1 & Flag-2 refers to the two flagellar systems found in E. coli. T2SS refers to the second type two secretion system (*yghJ-pppA-yghG-gspC-M*). ETT1 & ETT2 are the E. coli Type III secretion systems. EHS is the enterohemorrhagic type six secretion system, and ETSS2 is the Escherichia coli type six secretion system.

A key observation arising from the Group B1 comparison is that most virulence factors are found in LMEs outside the backbone genome (Additional File 2, Additional File 3, Additional File 4). For example, in the EHEC strains, the LEE is encoded on an LME, while shiga toxins are encoded on lambdoid phages; and in E24377A, the enterotoxin and CS3 fimbriae are encoded on plasmid pE24377A_79; and in 55989, the aggregative adhesion fimbrial operon is also plasmid-borne. While each strain had a number of lambdoid prophages present in its genome, only EHEC strains contained lambdoid prophages which encode the T3SS effectors which enhance virulence in these strains (Additional File 4). The presence of essential virulence factors on LMEs is consistent with previous findings, which have shown that non-pathogenic strains can be made pathogenic by introduction of elements found on LMEs [72,112]. Fitness factors related to colonisation of ecological niches not related to pathogenicity can also be found encoded on LMEs.

### Genome-scale reconstruction and metabolic profiling

GSMs are *in silico *metabolic models built using the collection of reactions that can be predicted from the annotated genome of an organism together with experimental data. They are used for many applications, including production strain design, examining evolutionary relationships, and linking phenotype and genotype information [113,114]. GSMs can be used to examine theoretical flux phenotypes, ATP maintenance, and redox balance requirements of cells under various genotypic and environmental conditions. These considerations allow prediction of growth rates and other characteristics such as organic acid production under specific conditions of interest. GSMs allow one to examine the effect of network alterations by performing *in silico *gene knock-out and gain-of-function experiments prior to labour-intensive and expensive wet-lab experiments. The first step in building a GSM is to reconstruct the metabolic network using the annotated genome (genome-scale reconstruction, GSR).

Numerous metabolic differences were observed between *E. coli *W and the other safe *E. coli *strains. In order to capture these differences, a GSR was constructed for *E. coli *W. Protein-coding genes from W were compared with those annotated in the *E. coli *K-12 MG1655 model, iAF1260 [115] using AUTOGRAPH [116]. Additional reactions were added or removed based on analyses of growth phenotypes, *in silico *simulations, and bibliomics (in-depth literature search). The resulting W model, iCA1273, includes 1,273 genes represented by 1,111 metabolites and 2,477 reactions (Additional File 5, Additional File 6). Relative to the K-12 model, iCA1273 is missing 41 genes that were not present in the W genome (Additional File 7). Conversely, iCA1273 contains 61 new genes, including 28 found in K-12 which had not previously been annotated (Additional File 8). Forty-eight genes found in the K-12 model, representing 155 reactions, were not included in iCA1273 as no functional orthologs were present in the W genome. In terms of modelling biomass formation, the most important difference between the two models was found in the production of membrane components. Fourteen genes involved in LPS synthesis in K-12 were not found in W and twelve LPS genes found in W were not found in K-12. Several genes common to both strains but not previously represented in the K-12 model were found. These included seven genes involved in the modification of LPS, specifically the inner core consisting of Kdo2-lipid A; two genes involved in the transport of peptidoglycan from the cytoplasm into the periplasmic space; and twelve genes involved the phenylacetic acid degradation pathway. Seven genes in the K-12 model were located on phage regions, whereas no genes encoding metabolic reactions relevant to the model were found in phage regions in the W genome. The localisation of gene-protein-reaction information was also refined relative to the K-12 model. Carbon and nitrogen source utilization were investigated using Biolog™ phenotype arrays (Additional File 9) in order to characterise the metabolism of the strain and further refine the GSR. All of these refinements allow improved resolution of pathways involved in metabolism in our model. Comparative analyses between K-12 and W were made both at genome and phenome levels [115,117] (Additional File 10). In addition, comparative studies were done between all four safe strains where appropriate. Key differences are detailed below.

#### Carbon and nitrogen source utilization

Sugars are ubiquitous throughout the environment and their breakdown supplies a key source of carbon and energy for bacteria. Sucrose is the main carbohydrate transport molecule in plants, and is therefore the most abundant disaccharide encountered in most environments. A key metabolic difference between *E. coli *W and the other three safe strains is the ability of *E. coli *W to grow on sucrose. This is due to the presence of the *csc *regulon, which was originally described in *E. coli *EC3132 and encodes a regulator (*cscR*), a sucrose transporter (*cscB*), an invertase (*cscA*) and a fructokinase (*cscK*) [118]. The *csc *regulon has been inserted between the highly variable *argW *gene region and the *dsdX *gene of the D-serine regulon [119,120]. Due to the insertion in *dsdX*, a D-serine transporter, *E. coli *W has lost the ability to utilize D-serine.

Several operons have been identified in *E. coli *strains for uptake and metabolism of cellobiose, a glucose disaccharide formed by hydrolysis of cellulose. The four safe strains contain only the six gene *bgl *regulon for cellobiose metabolism. This operon has been reported to be silenced in wild-type *E. coli *strains [121] and K-12 is unable to grow on cellobiose [122]. In contrast, W displays weak growth on cellobiose, indicating that the *bgl *genes are not silenced. Uptake of the β-glycosides salicin and arbutin is generally seen in conjunction with cellobiose uptake [122], though *E. coli *W exhibited growth only on salicin. The absence of the arbutin transporter gene *arbT *[122] is the most likely explanation for lack of growth on arbutin.

The pentose monosaccharide D-ribose is a key component of DNA and RNA; D-allose is a ribose analog. Ribose can be transported into the cell [123] and enter amino acid and pentose phosphate pathways after it is phosphorylated; allose can be converted to fructose-6-phosphate [124] for entry into central carbon metabolism. The D-allose transporter can also transport D-ribose [125]. In contrast to the other safe strains, W is unable to catabolise ribose or allose; this is explained by the absence of the *rbsDACBKR *[123,124] and *alsBACEK *[125] regulons in W.

Many environmental applications require industrial strains to break down aromatic compounds, which are typically found in soil and water. This capability varies between safe strains. W is able to break down the widest range of aromatic compounds among four strains [17]. Unlike the other strains, K-12 is unable to break down 3- and 4-hydroxyphenylacetic acids as it does not contain the eleven-gene *hpa *gene cluster [17].

Both K-12 and W can break down phenylacetic acid due to the presence of *paa *gene cluster. *E. coli *B has lost this cluster due to an IS*3*-mediated insertion while Crooks has an intact *paa *gene cluster and can presumably also break down phenylacetic acid. *E. coli *W was isolated from soil, which may help explain its capability to break down diverse aromatic compounds. In addition, loss of extraneous carbon source genes can be observed in strains maintained for long periods on laboratory carbon sources [127]. Since W was archived shortly after isolation, it is less likely to have undergone this selective pressure.

D-Galactosamine is a constituent of animal glycoprotein hormones while *N*-acetyl-D-galactosamine (NAG) is a core component of peptidoglycan. Both are important nitrogen sources. W shares with B and Crooks the *agaV-I *gene cluster, which is involved in D-galactosamine and NAG catabolism [128,129]. This cluster has been partially lost in K-12 due to deletion of *agaEF*.

In K-12, two separate base pair insertions in *ilvG *result in valine sensitivity [130]. When K-12 is grown with valine as a nitrogen source, valine accumulation results in positive inhibition of the branched chain amino acid synthesis pathway and a subsequent deficit of isoleucine and leucine. *IlvG *is intact in W, B and Crooks; consequently, these strains are likely to have high L-valine tolerance.

There are a number of discrepancies between model predictions and phenotype array data (Additional File 10). In some cases, C and N sources which can be used by W and K-12 according to the phenotype array data are not supported by model predictions. This can be explained by insufficient annotation of metabolic pathways for many of these C and N sources. In other cases, the models predict utilization of C and N sources which do not support growth (or support only poor growth) in phenotype arrays; in these cases, it is likely that specific conditions (e.g. anaerobic growth, requirement for cofactors) are not met in the phenotype assay.

#### Other metabolic considerations

Inorganic ions such as iron and cobalt play important roles in many biological processes, and there are many uptake systems available for different ionic forms. W differs from other safe strains in two ion transport systems. Firstly, it does not contain the seven-gene *tonB*-dependant diferric dicitrate uptake system, *fecIRABCDE*. In K-12 and B, this gene cluster is located within the phage-like element KpLE2. Secondly, it has a cobalt transport system, *cbiQ-O2*, located in the region *epd-yggC*; this transport system is not present in the other three strains.

## Conclusions

*E. coli *W has been used in research laboratories and for industrial applications for almost seventy years. Because of this long history, the strain is considered a 'safe' laboratory strain. The safety of a strain is an important consideration both for laboratory research and for industrial applications. Containment and handling in both environments is less complex for safe strains, and safety requirements can significantly impact on the economics of production. Like other safe strains, W harbors genes which encode pathogenicity determinants. W has more such genes than other safe strains; however, many have been mutationally inactivated or are missing key components required for pathogenicity. These observations confirm the historical attribution of W as a safe strain.

Amongst the four safe laboratory strains, W has several unique features: it belongs to phylogroup B1 rather than A; it has a larger genome size; and the period of time between isolation and strain archiving was relatively short. The two latter features are probably related: strains that are maintained under laboratory conditions for extended time periods are subject to specific selection pressures, and tend to lose genes which are not required for survival under laboratory conditions [127]. In line with this, and consistent with its larger genome size, the W genome encodes more genes than other safe strains. Additionally, it has fewer ISs, which tend to multiply in genomes of organisms maintained under laboratory conditions [131]. Overall, W is more similar to other pathogenic and commensal strains than it is to the other safe laboratory strains. Furthermore, it has the largest backbone sequence of the Group B1 strains, suggesting that it has the most complete complement of ancestral genes. These considerations place W as the preferred laboratory strain for use in genomic comparisons aimed at investigating genes involved in pathogenicity and commensalism.

Like other wild-type isolates [132], W encodes a large number of carbon source utilization genes, and it grows on a much broader range of carbon substrates than K-12 strains (Additional File 9). Of particular interest is the ability of W to utilize sucrose as a carbon source. For industrial production applications, in particular for large-scale production of commodity biochemicals (e.g., biofuels, industrial polymers, and other industrial feedstocks), sucrose from sugarcane is the preferred carbon source [29]. It is abundant, it is cheaper than glucose [133] and it is also 'greener' than glucose; for example, greenhouse gas emissions for ethanol production are reduced by 85% relative to petrochemicals when using sugarcane sucrose as a carbon source, whereas use of glucose from corn reduces emissions by only 30% [133]. The growth of W on sucrose, in combination with its many other desirable industrial traits (fast growth rate, growth to high cell densities, lack of adhesins which result in clumping, lack of antibiotic markers, and relative resistance to environmental stresses) also place *E. coli *W as a preferred strain for industrial biotechnology applications. Some of these characteristics (e.g. sucrose utilisation and lack of adhesins/antibiotic markers) are easily explained by genome analysis. However, the raw sequence data does not shed any light on why W exhibits the other characteristics. Further experimental analysis using a systems biology approach might shed light on this.

An annotated genome sequence is an important step in characterisation of an organism, and allows construction of genome scale models which can be used to (a) interrogate the metabolic attributes of organisms and (b) facilitate strain development for industrial applications. Our W GSR includes a number of genes which were not annotated in the original K-12 GEM; this includes both genes that are unique to W and genes that were omitted from the K-12 model. Our improved model more accurately reflects the metabolism of an *E. coli *cell. There is good agreement between genome data, phenome data, and model data; the combination of these allows us to define the metabolic capabilities of *E. coli *W both *in vitro *and *in silico*. The W strain exhibits many industrially desirable traits, including fast growth, stress tolerance, growth to high cell densities, and the ability to utilise sucrose efficiently [22,24-28]. With the availability of an annotated genome and GSR, the W strain can now be used as a platform organism for developing sucrose-based bioprocesses to replace current unsustainably-produced industrial chemicals.

## Methods

### Sequencing and assembly

*E. coli *W (ATCC 9637) was obtained from NCIMB Ltd (Aberdeen, Scotland; Accession Number 8666. The NCIMB stock was supplied by ATCC). Roche/454 pyrosequencing and fosmid end sequencing followed by manual gap-filling were used to construct the *E. coli *W genome. The shotgun reads in SFF files that were produced from GS 20 (707,210 reads, 81.8 Mb; MWG Biotech, Germany) and GS FLX (236,190 reads, 56.5 Mb; National Instrument Center for Environmental Management, Korea), totalling *ca. *27.7× genome coverage, were assembled into 209 contigs by Roche's gsAssembler. CONSED [134] was used for sequence manipulation that included read/contig editing, primer design, and finish read processing. Specifically, 127 large contigs with accompanying quality scores produced by the gsAssembler were imported into CONSED as single-read contigs. 2,479 paired-end reads of pCC1FOS (EPICENTRE Biotechnologies, United States) off from ABI 3700 (1.98 Mb, ca. 9.9× clone coverage; GenoTech Co., Korea) were then aligned on the contigs and the resulting scaffolds were validated using the mate information derived from the fosmid end reads.

The remaining sequence gaps were filled by Sanger sequencing of the gap-spanning PCR products or fosmid clones. Repeat-induced over-collapsed short contigs were resolved by reproducing contigs according to the copy number deduced from the read depth of contigs and by ordering them using 'from/to' information given by the gsAssembler. The most difficult assembly was with two highly similar copies of P2-like prophages (31,005 bp and 32,732 bp); each was reconstructed into the relevant sequences after disentangling the over-collapsed contigs. Ambiguous sequences resulting from the differences of the two prophages were refined by primer walks on fosmid clones containing each prophage segment. The overall error rate of the assembled genome sequence was calculated as 0.09 bp/10 kb, and verification of the assembly came from the consistency of fosmid end reads on the final contig.

The sequence was validated by comparison against independent sequence data generated using a GAII platform. The 65-bp reads were assembled by scaffolding against the original sequence using Burrows-Wheeler Aligner (BWA) [135]. SNPs and INDELS relative to original sequence were identified using SAMtools [136]. Corrections were made based on confidence (related to depth of local sequencing) for each reported discrepancy.

### Annotation

ORF prediction was performed using Prodigal [32] and Glimmer [33]. AutoFACT [137], an automatic annotation pipeline, was employed to score predicted ORFs against existing databases, including non-redundant protein sequences (nr) in GenBank [138], KEGG [139] and COG [140], using homology search. Where the AutoFACT annotation differed from the K-12 annotation for shared orthologs, the difference was resolved through manual curation. In particular, if AutoFACT proposed a less ambiguous annotation, experimental evidence for the AutoFACT annotation was sought in the literature. tRNA genes were predicted using tRNAscan-SE [141], rRNA genes were predicted using rnammer [142], and ncRNA genes were predicted using INFERNAL [143]. These predictions were integrated into the annotation using Artemis [144]. ORFs which resided within rRNA genes and ncRNAs covering rRNA or tRNA genes were removed. Transcriptional start sites were further curated using Artemis and modified based on matches to homologous genes from *E. coli *K-12, B and Crooks. CRISPR regions were predicted using a combination of CRT [145] and PILER [146].

### Comparative Genome Analysis

Comparative genome analysis was based on protein-coding sequences predicted from the *E. coli *W (ATCC 9637) annotation and three other safe *E. coli *strains: K-12 MG1655 [GenBank:U00096], B REL606 [GenBank:CP000819], and Crooks ATCC 8739 [GenBank: CP000946]. Comparative analysis of the *E. coli *W plasmids pRK1 and pRK2 was based on protein-coding sequences and was performed against five representative plasmids: pSE11-1 [GenBank: AP009241], pSE11-3 [GenBank: AP009243], ColIb-P9 [GenBank:AB021078], R64 [GenBank:AP005147, and pSE11-5 [GenBank: AP009245]. All-against-All BLASTP for amino acids was used to assign orthologs; these were further curated using gene context data, analysis of orthologs provided by the *E. coli *B REL606 genome annotation, and literature data.

Protein-coding genes and pseudogenes were mapped to orthologs in each of the three other sequenced laboratory strains by BLAST to attain the bi-directional best hit (BBH) relationships. Genes with high sequence similarities to a gene in another strain but differing significantly in length were inspected manually to establish the cause of variation.

Insertion Sequences (ISs) for *E. coli *W, Crooks and SE11 were annotated using BLASTN against the ISFinder database [147,148]. Large mobile elements and rearrangement hot spot (Rhs) elements were identified during the annotation using BLASTP against the nr database in GenBank. Labels for *rhs *genes were assigned using nomenclature described by Jackson et. al. (2009).

Phylogenetic analysis was performed using the gene concatenation method [36]. Concatenated sequences of seven housekeeping genes (*adk, fumC, gyrB, icd, mdh, purA, recA*) and sequence types (STs) of *E. coli *reference (ECOR) collection strains and related organisms were downloaded from the *E. coli *MLST Database [149]. W gene sequences were aligned using ClustalW [150] then concatenated. A phylogenetic tree was generated by the neighbour joining method with 1000 bootstrap iterations using MEGA4 [151].

### Motility Assay

Motility assays was performed as described previously [95] with the following alterations: assays were performed at 25°C and 37°C only, and antibiotics were not included in the medium.

### GSR Construction

The GSR was created using AUTOGRAPH [116] to generate a database of predicted ORFs against the *E. coli *K-12 GSR, iAF1260 [115]. Additional reactions were added or removed based on an in-depth literature search, high-throughput carbon/nitrogen/phosphorous/sulphur source growth assays (PM Kit, Biolog, Hayward, CA) and *in silico *validation using the COBRA toolbox [152] to ensure all biomass components could be synthesized. *In silico *simulations used the biomass composition of iAF1260 [115].

Gene-protein-reaction associations were curated and assigned a confidence score based on experimental data and information from the *E. coli *K-12 iAF1260 GEM. Boolean logic was employed to denote the relationships between proteins and whether they formed complexes; isozymes were described as an 'OR' relationship and protein complexes were represented as 'AND' relationships linked to other peptides required for a functional protein. In cases where different combinations of proteins can form a complex which catalyses the same reaction, each complex was represented by an 'AND' relationship and 'OR' relationships were made between complexes. Gaps in the metabolic network, resulting from missing genes which are essential for the synthesis of biomass components and production of waste products, were filled by incorporating reactions from the iAF1260 and KEGG database.

## List of abbreviations

BBH: bi-directional best hit; CAS: CRISPR associated sequence; COG: clusters of orthologous groups of proteins; CPS: capsular polysaccharide; CRISPR.: clustered regularly interspaced short palindromic repeat; ECOR: *Escherichia coli *Reference Collection; EHS: enterohaemorrhagic *E. coli *type six secretion system cluster; ELF: *E. coli *YcbQ laminin-binding fimbria; ETEC: enterotoxigenic *E. coli*; ETT1: *E. coli *Type III secretion system 1; ETT2: *E. coli *Type III secretion system 2; GEM: genome-scale model; GSR: genome-scale reconstruction; HGT: horizontal gene transfer; H-rpt: H-repeat; IncI1: Incompatability group I1; IS: insertion sequence; KEGG: Kyoto Encyclopaedia of Genes and Genomes; LME: large mobile element; LPS: lipopolysaccharide; NAG: *N*-acetyl-D-galactosamine; ORF: open reading frame; PGA: penicillin G acyclase; pLE: phage-like element; Rhs: rearrangement hot spot; T2SS: type II sectrtion system; T3SS: type III secretion system; T6SS: type VI secretion system; UPEC: uropathogenic *E. coli*; WpLE: *E. coli *W phage Like Elements

## Authors' contributions

LKN and SYL conceived the idea for the project. LKN and CEV were responsible for project management and supervision. Genome sequencing and automated annotation was performed by JFK and HJ. CTA did the manual curation of the annotation, comparative anlayses, and genome scale reconstruction. CEV, CTA and LKN wrote the manuscript. All authors contributed to revision of the manuscript. All authors have read and approved the final manuscript.

## Supplementary Material

Additional file 1 **List of CDSs which occur once in the genome of one safe strain but more than once in genomes of other safe strains**. A list of CDSs which have only one copy in one safe strain, but have more than one ortholog in one or more other safe strains. For example, *hokE *occurs once in the K-12 genome but multiple times in the W genome. The CDS count of each strain does not reconcile unless these one-to-many and many-to-many relationships are considered. Detailed CDS counts are provided within the file. The counts explain the CDS skew which occurs when counting the number of CDSs in Figure 2 for K-12, B, or ATCC 8739. For example, in ATCC 8739 one copy of EcolC_3064 is present, while two are present in W as ECW_m0635 and ECW_m0636. When shared orthologs are counted the number in the ATCC 8739-W region can be one or two, depending on whether the number of orthologs is taken from W or ATCC 8739s context. We have thus detailed all orthologous CDSs which are found in different copy numbers in the other safe strains genomes. Click here for file

Additional file 2 **Description of supplementary files and instructions for use thereof**. Detailed description of the contents of each additional file. Click here for file

Additional file 3 **Plasmids found in Group B1 strains**. Overview and analysis of the integrative elements which are present in each sequenced group B1 strain. Sheet "Group B1 IEs" presents the attachment sites and significant fitness or virulence factors which are present in each integrative element. Sheet "IE sizes" shows the assumed start and finish sites of each integrative element and the elements size. These sizes were used to calculate each group B1 strains genome backbone size. Click here for file

Additional file 4 **Integrative elements found in Group B1 strains**. Analysis of the plasmids which are found in sequenced group B1 strains including plasmid size and fitness/virulence factors which are present on each plasmids genome. Click here for file

Additional file 5 **iCA1273 GSR**. A list of the reactions, including GPR associations and constraints (lower bound, upper bound, objective functions) which are present in iCA1273. Click here for file

Additional file 6 **iCA1273 GSR**. iCA1273 in xml format for use with the COBRA Toolbox. Click here for file

Additional file 7 **List of unique iAF1260 features compared to iCA1273**. A list of reactions which are present in iAF1260 but either do not occur in iCA1273 or do occur but have different gene-protein-reaction associations. Data columns are as follows: 1. Reaction abbreviation 2. Function of the reaction 3. Reaction catalysed 4. The genes necessary for the reaction to be catalysed in Boolan format 5. Notes about the reaction including reference to literature which details experimental evidence for the reaction and the PubMed ID of the paper. Click here for file

Additional file 8 **List of unique iCA1273 reactions and metabolites compared to iAF1260**. A list of new reactions and metabolites in iCA1273 which are not found in iAF1260. This file contains the following: 1. "Missing iAF1260 reactions" details reactions which occur in iAF1260 that are not present in W 2. "iCA1273 rxns miss K12 ortho" details reactions from iAF1260 which still occur in iCA1273 but are missing genes which are not present in the W genome. e.g. reaction "RPE" from iAF1260 can be catalyzed by the enzyme encoded by b3386 or b4301. However, in W, an ortholog for b4301 is not present while an ortholog for b3386 is present so the reaction still occurs within the cell. Click here for file

Additional file 9 **Growth phenotype data for E. coli W (ATCC 9637)**. Results of the Biolog™ growth phenotype assays for *E. coli *W and *E. coli *K-12 on a wide range of carbon and nitrogen sources. Click here for file

Additional file 10 **Comparison between predictions and experimental growth data for K-12 GEM and W GSR**. A comparison between K-12 GEM (iAF1260) predicted growth phenotypes and Biolog™ data growth, and between W GEM (iCA1273) predicted growth phenotypes and Biolog™ data growth. Overlap between predicted and actual growth phenotypes is higher in W than in K-12. Click here for file
