# Supplementary material for: The genome sequence of E. coli W (ATCC 9637): comparative genome analysis and an improved genome-scale reconstruction of E. coli
Source: BMC Genomics. 2011 Jan 6;12:9. doi: 10.1186/1471-2164-12-9 (PMC3032704; doi:10.1186/1471-2164-12-9)
Supplement: Additional file 2 — Description of supplementary files and instructions for use thereof. Detailed description of the contents of each additional file. [file 1471-2164-12-9-S2.DOC]

**List of Supplementary Files**

This file details the contents of each Supplementary File and how to use it.

**Supplementary File 1 – Description of supplementary files** **and instructions for use thereof**

This file

**Supplementary File 2 - Integrative elements found in Group B1 strains**

Overview and analysis of the integrative elements which are present in each sequenced group B1 strain. Sheet “Group B1 IEs” presents the attachment sites and significant fitness or virulence factors which are present in each integrative element. Sheet “IE sizes” shows the assumed start and finish sites of each integrative element and the elements size. These sizes were used to calculate each group B1 strains genome backbone size.

**Supplementary File 3 - Plasmids found in Group B1 strains**

Analysis of the plasmids which are found in sequenced group B1 strains including plasmid size and fitness/virulence factors which are present on each plasmids genome.

Supplementary File 4 - iCA1273 GSR (XLS Format)

A list of the reactions, including GPR associations and constraints (lower bound, upper bound, objective functions) which are present in iCA1273

Supplementary File 5 - iCA1273 GSR (XML format)

iCA1273 in xml format for use with the COBRA Toolbox

Supplementary File 6 - List of unique iAF1260 features compared to iCA1273

A list of reactions which are present in iAF1260 but either do not occur in iCA1273 or do occur but have different gene-protein-reaction associations. Data columns are as follows:

1. Reaction abbreviation

2. Function of the reaction

3. Reaction catalysed

4. The genes necessary for the reaction to be catalysed in Boolan format

5. Notes about the reaction including reference to literature which details experimental evidence for the reaction and the PubMed ID of the paper

Supplementary File 7 - List of unique iCA1273 reactions and metabolites compared to iAF1260

A list of new reactions and metabolites in iCA1273 which are not found in iAF1260. This file contains the following:

1. "Missing iAF1260 reactions" details reactions which occur in iAF1260 that are not present in W

2. "iCA1273 rxns miss K12 ortho" details reactions from iAF1260 which still occur in iCA1273 but are missing genes which are not present in the W genome. e.g. reaction "RPE" from iAF1260 can be catalyzed by the enzyme encoded by b3386 or b4301. However, in W, an ortholog for b4301 is not present while an ortholog for b3386 is present so the reaction still occurs within the cell.

Supplementary File 8 – Growth phenotype data for *E. coli* W

Results of the Biolog^TM^ growth phenotype assays for *E. coli* W and *E. coli* K-12 on a wide range of carbon and nitrogen sources

Supplementary File 9 – Comparison between predictions and experimental growth data for K-12 GEM and W GSR

A comparison between K-12 GEM (iAF1260) predicted growth phenotypes and Biolog^TM^ data growth, and between W GEM (iCA1273) predicted growth phenotypes and Biolog^TM^ data growth. Overlap between predicted and actual growth phenotypes is higher in W than in K-12.

Supplementary File 10 - List of CDSs which occur once in the genome of one safe strain but more than once in genomes of other safe strains

A list of CDSs which have only one copy in one safe strain, but have more than one ortholog in one or more other safe strains. For example, *hokE* occurs once in the K-12 genome but multiple times in the W genome. The CDS count of each strain does not reconcile unless these one-to-many and many-to-many relationships are considered. Detailed CDS counts are provided within the file. The counts explain the CDS skew which occurs when counting the number of CDSs in Figure 2 for K-12, B, or ATCC 8739. For example, in ATCC8739 one copy of EcolC_3064 is present, while two are present in W as ECW_m0635 and ECW_m0636. When shared orthologs are counted the number in the ATCC 8739-W region can be one or two, depending on whether the number of orthologs is taken from W or ATCC8739s context. We have thus detailed all orthologous CDSs which are found in different copy numbers in the other safe strains genomes.
